# PFBNet: a priori-fused boosting method for gene regulatory network inference

**DOI:** 10.1186/s12859-020-03639-7

**Published:** 2020-07-14

**Authors:** Dandan Che, Shun Guo, Qingshan Jiang, Lifei Chen

**Affiliations:** 1 Shenzhen Key Lab for High Performance Data Mining, Shenzhen Institutes of Advanced Technology, Chinese Academy of Sciences, Shenzhen, 518000 China; 2 School of Mathematics and Computer Science, Fujian Normal University, Fujian, 350117 China

**Keywords:** Gene regulatory network inference, Time-series expression data, Boosting, Prior information fusion

## Abstract

**Background:**

Inferring gene regulatory networks (GRNs) from gene expression data remains a challenge in system biology. In past decade, numerous methods have been developed for the inference of GRNs. It remains a challenge due to the fact that the data is noisy and high dimensional, and there exists a large number of potential interactions.

**Results:**

We present a novel method, namely priori-fused boosting network inference method (PFBNet), to infer GRNs from time-series expression data by using the non-linear model of Boosting and the prior information (e.g., the knockout data) fusion scheme. Specifically, PFBNet first calculates the confidences of the regulation relationships using the boosting-based model, where the information about the accumulation impact of the gene expressions at previous time points is taken into account. Then, a newly defined strategy is applied to fuse the information from the prior data by elevating the confidences of the regulation relationships from the corresponding regulators.

**Conclusions:**

The experiments on the benchmark datasets from DREAM challenge as well as the *E*.*coli* datasets show that PFBNet achieves significantly better performance than other state-of-the-art methods (Jump3, GEINE3-lag, HiDi, iRafNet and BiXGBoost).

## Background

In system biology, comprehending the intricate gene regulatory network (GRN) is of significant important, since it provides insights to understand the cell physiology, development and pathogenesis [1, 2]. With the advent of high-throughput experimental techniques such as RNA-Seq and DNA microarrays, inferring the GRN from such data at genomic scale is feasible. However, it is still a challenge due to the high-dimensional and noisy characteristics of the data, and the regulatory network may be obscured by the indirect connections. Another problem is that the samples of the data are often relatively few compared to the number of genes (i.e., the *n*≪*p* problem [3]). So far, various methods have been developed for inferring GRNs from expression data, including Bayesian Networks-based methods [4–9], information theory-based methods [6, 10–15], Ordinary Differential Equation (ODE) based methods [16–19], ensemble framework based methods [20–25], etc. Here we briefly review some algorithms that are related to our work. Among these approaches, the algorithms that under the ensemble framework have emerged as the strong players, such as GENIE [22], TIGRESS [21], BiXGBoost [25], etc. The key idea of the ensemble framework is to decompose the GRN inference problem into *p* feature selection subproblems (*p* is the number of genes in the data) and solve each subproblem with the corresponding regression model. As the regression model is selected, the confidences of the regulation relationships that from each candidate regulator (i.e., transcription factors (TFs)) to the corresponding target gene could be calculated as the feature weight. Finally, outputs from each subproblem are fused to reconstruct the GRN. Several algorithms (e.g., TIGRESS) chose the linear model to address the problem, however, they may not perform well if the data presents a higher-order structure. On the other hand, the algorithms that utilizing the nonlinear model can easily be computationally intractable as the number of the candidate regulators increase remarkably. Although these algorithms are successful, they inferred the GRN only used a single type of data (i.e., the gene expression), whereas other types of data (e.g., expression from the knockout) may provide non-redundant information about the directionality of regulatory relationships [23]. To this end, it is important to incorporate the prior information (e.g., the information from the knockout data) in GRN inference, which may lead the GRN to be more reliable and interpretable.

Compared with the steady-state expression data, time-series expression data are more helpful to identify the regulation relationships that reflect the temporal dynamics [26]. In this regard, many algorithms have been developed to address these data [10, 15, 16, 23, 25, 27, 28], representative algorithms including GENIE-lag [27], Jump3 [28], BiXGBoost [25], etc. For most of the available algorithms, one common practice to tackle the time-series data is building the model under the assumption that the target gene expression at current time point is simply affected by the expressions of the regulators at previous time point. Clearly, the information of the candidate regulators at earlier time points would be ignored for these methods, which may affect the accuracy of the inferred GRN. The recent method BiXGBoost considers the candidate regulators at many time points that would affect the target gene, and selects the time point with most impact, where it showed promising performance compared with the traditional methods. However, impact of the regulation is more likely to be the accumulation of previous time points rather than the maximal one. Moreover, as mentioned before, another limitation for these algorithms is that they have no mechanism for integrating the prior information from other types of data.

Recently, iRafNet [23] was introduced, which integrated different types of data via calculating the corresponding weights under the Random Forest (RF) framework. It utilized the prior information in data pre-processing stage and outperformed the original RF-based method as well as the community learning algorithm on benchmark datasets. Yue Deng et al. [16] proposed an Ordinary Differential Equation (ODE) based method named HiDi, which formulated the prior knowledge as a constrained optimization problem. It filtered out impossible regulatory relationships by exploring the prior information with the outlier detection techniques. HiDi showed superior performance over other algorithms on DREAM4 challenge. However, one limitation of ODE based method is that the linearity assumption is made on the gene regulations, which may not be quite consistent with the real regulations.

**Fig. 1:**
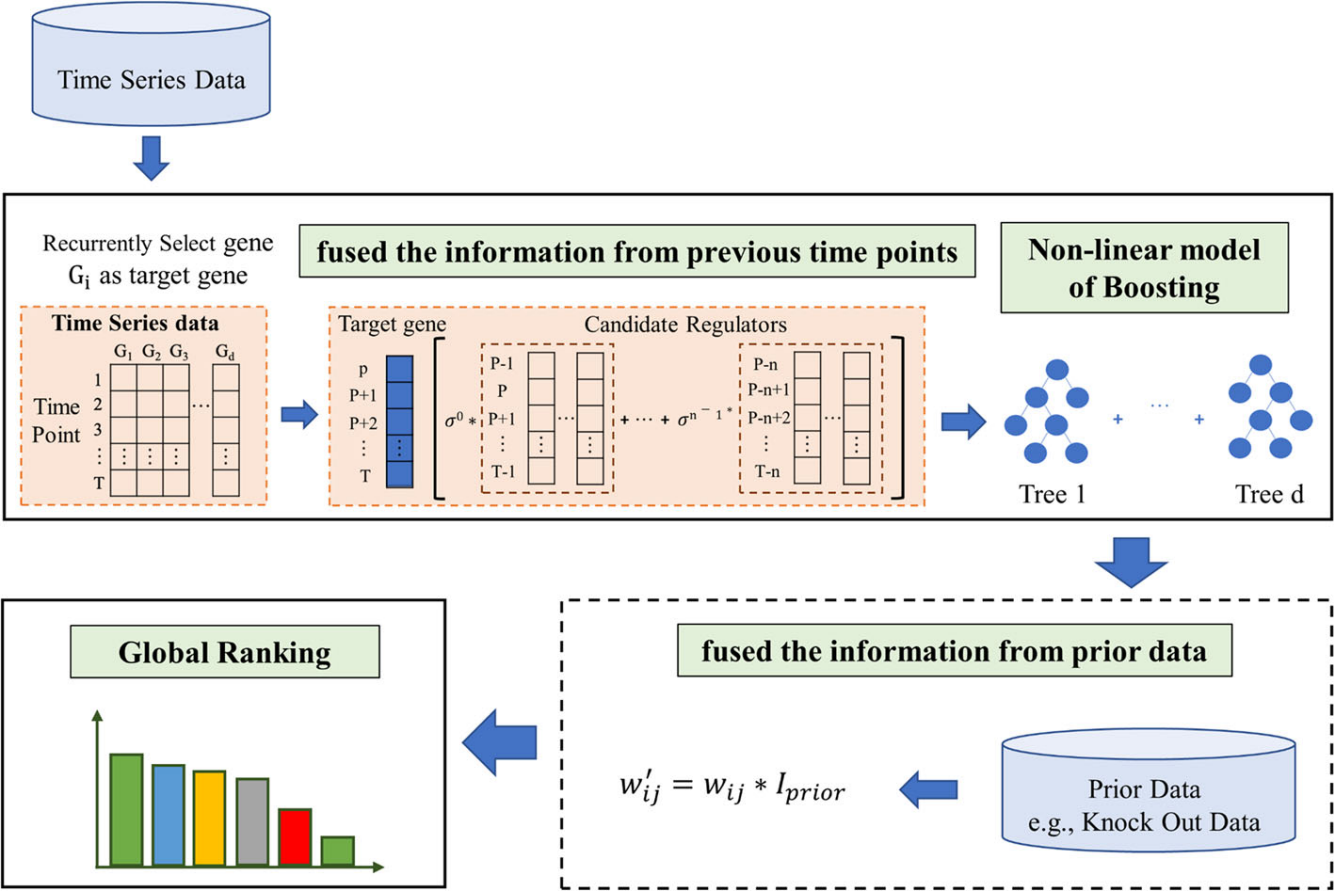
The schematic diagram of PFBNet. PFBNet recurrently selects one gene as the target gene, and construct the feature selection subproblem that the information from previous time points is fused; then, the non-linear model of boosting is applied to solve the subproblem; subsequently, the prior information is fused into the model and the GRN is inferred according to the global ranking of the confidences of the regulation relationships

To overcome these limitations, here, we propose PFBNet, a new approach to infer the GRN from time-series expression data. The schematic diagram of our algorithm is shown in Fig. 1. Specifically, PFBNet fuses the information of candidate regulators at previous time points base on the non-linear model of boosting; then, the prior information is fused into the model via recalculating the weights of the corresponding regulation relationships. To demonstrate the performance of our method, we apply it on the widely used benchmark datasets from DREAM [29] challenge as well as the *E*.*coli* datasets [30] for comparing various GRN inference algorithms. The results show that our algorithm outperforms other state-of-the-art algorithms (i.e., Jump3, GEINE3-lag, HiDi, iRafNet and BiXGBoost).

## Results

### Datasets

We evaluate the performance of our algorithm PFBNet on the benchmark datasets from DREAM4 *in*-*silico* size 100 challenge [29] as well as the *E*.*coli* datasets [30]. The dataset from DREAM4 *in*-*silico* size 100 challenge contains five networks with 100 genes, where time-series expression data and knockout data are provided for each of them. Specifically, the time-series expression data involves 10 samples with 21 time points; the knockout data includes the gene expression with knocking out each one of the 100 genes. The *E*.*coli* datasets provide the time-series expression data corresponding to different environmental conditions. The datasets under three environmental conditions (i.e., cold, heat and oxidative stress) are chosen as our experimental datasets. The gold standard of the datasets comes from the DREAM5 challenge [31] and the experimental verification of RegulonDB [32]. We preprocessed the *E*.*coli* datasets in the light of [25], and retained 163 transcription factors (TFs) as well as 1484 target genes for evaluation. The details of the datasets are summarized in Table 1.

**Table 1:** The details of the datasets

Network	#Genes	#Candidate regulators	#samples	#Time points	#edges
DREAM4 *in*-*silico* size 100 Network 1	100	100	10	21	176
DREAM4 *in*-*silico* size 100 Network 2	100	100	10	21	249
DREAM4 *in*-*silico* size 100 Network 3	100	100	10	21	195
DREAM4 *in*-*silico* size 100 Network 4	100	100	10	21	211
DREAM4 *in*-*silico* size 100 Network 5	100	100	10	21	193
*E*.*coli* cold Networks	1484	163	3	8	3080
*E*.*coli* heat Networks	1484	163	3	8	3080
*E*.*coli* oxi Networks	1484	163	3	11	3080

### Evaluation metrics

To evaluate the performance of our algorithm, two widely used evaluation metrics, AUROC (the area under the receiver operating characteristic curve) and AUPR (the area under the precision-recall curve) are considered. Specifically, we computed TP (the number of true positives), TN (the number of true negatives), FP (the number of false positives) and FN (the number of false negatives). Then, *TPR*(*TPR*=*TP*/(*TP*+*FN*)) and *FPR*(*FPR*=*FP*/(*FP*+*TN*)) can be calculated, which are the horizontal and vertical coordinates of the receiver operating characteristic curve respectively. Based on this, AUROC can be obtained. Similarly, AUPR can be calculated according to the corresponding Precision (*TP*/(*TP*+*FP*)) and Recall (*TP*/(*TP*+*FN*)).

### Parameters setting of PFBNet

The boosting model XGBoost is applied in our algorithm, where the python package of XGBoost provides various parameters for implementation. We choose the decision tree as the base learner since it is non-linear. Similar with other algorithms (e.g., BiXGBoost), these parameters were confirmed in practice.We found that most of these parameters were not sensitive to the performance of the algorithm. The parameters *max*_*depth* and *min*_*child*_*weight* are related to the structure of each tree in the model and they are both set to 4. The parameter *subsample*, which controls the ratio of the training samples in each tree, is set to 0.7. The parameter *colsample*_*bytree* controls the ratio of features (candidate regulators) in each tree, and is set to 0.9 here. The learning rate *eta* is set to 0.0008. The number of trees is set to 1000, where it is the same default as most ’tree-based’ methods (e.g., iRafNet and BiXGBoost). More details of parameters selection are available on Supplementary data (see Additional Fig S1-S6).

**Fig. 2:**
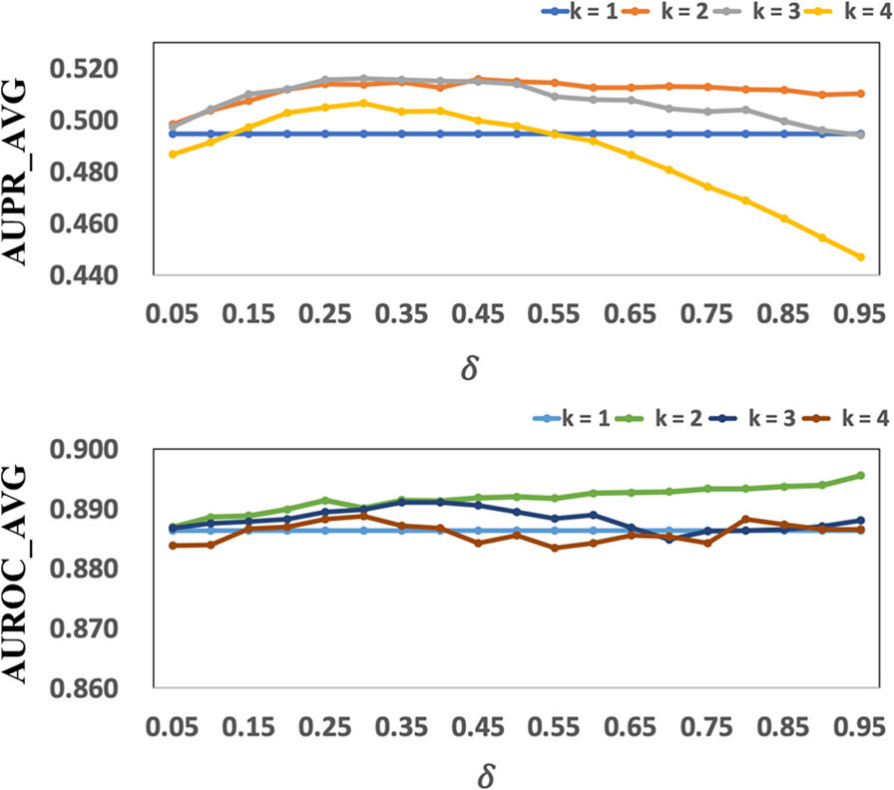
Effects of parameters *k* and *δ* for PFBNet on DREAM4 ***in***-*silico* size 100 challenge networks. The AUPR_AVG and AUROC_AVG are the average AUPR and AUROC scores respectively for the five networks

Two newly defined parameters (i.e., *k* and *δ*) are set to 2 and 0.45 respectively in this study. Specifically, *k* is the number of previous time points for all regulators that the related information is considered to fused in our model (Table S1) and *δ* is the decay factor that reduced the influence of the candidate regulators from the earlier time point on the target gene (see Eq. 5). Figure 2 shows the effects of these parameters on the performance of our PFBNet algorithm in terms of AUPR and AUROC. We found that the averaged AUPR first increased and then decreased along with the increasing of *δ* as *k*>1. And the averaged AUROC kept increasing with the *δ* as *k*=2.

### Performance evaluation on simulation datasets

The DREAM4 *in*-*silico* size 100 challenge networks with time-series data were used to asses PFBNet algorithm, and several state-of-the-art GRN inference algorithms including GENIE-lag [27], Jump3 [28], BiXGBoost [25], iRafNet [23], HiDi [16] and the winner of the DREAM challenge were chosen for comparison.

GENIE-lag, Jump3 and iRafNet are all random forest (RF) [33] based algorithms, while Jump3 integrates the natural interpretability of differential model from time-series expression data. BiXGBoost fuses the information of the candidate regulators at the time point with most impact and integrates XGBoost model to reconstruct the GRN. iRafNet and HiDi both utilize the prior information to improve the accuracy of GRN inference. The parameters of all these algorithms were set to default values for a fair comparison. The averaged AUPR and AUROC were chosen as the criteria in the experiments.

Since GENIE-lag, Jump3 and BiXGBoost have no mechanism for integrating the information from prior data, for a fair comparison, the PFBNet was implemented without fusing the prior information. Table 2 shows the comparative results of these algorithms on the datasets from DREAM4 *in*-*silico* size 100 challenge. As it is shown that, PFBNet achieves best performance compared with other methods in terms of both AUPR and AUROC for all the five networks. Specifically, for each network, PFBNet shows **31%** to**57%** improvements (Network1: 32%, Network2: 38%, Network3: 31%, Network4: 48% and Network5: 57%) than the second-best algorithm in terms of AUPR. Meanwhile, PFBNet achieves 3.1%, 5.2%, 3.8%, 5.8% and 4.1% improvements than the second-best algorithm in terms of AUROC for the five networks respectively. Clearly, the improvement of PFBNet in terms of AUPR is much more impressive than that in terms of AUROC. And it should be noted that, since most of GRNs are sparse, the AUPR is more meaningful than AUROC[28].

**Table 2:** Comparison of different methods on the DREAM4 *in*-*silico* size 100 challenge networks (without utilizing the information from prior data)

**Method**	**GENIE-lag**		**Jump3**		**BiXGBoost**		**PFBNet**	
**Data used**	TS		TS		TS		TS	
**Metrics**	AUPR	AUROC	AUPR	AUROC	AUPR	AUROC	AUPR	AUROC
**Network_1**	0.183	0.791	0.270	0.772	0.235	0.806	**0.360**	**0.831**
**Network_2**	0.109	0.708	0.110	0.665	0.152	0.730	**0.210**	**0.768**
**Network_3**	0.224	0.765	0.200	0.741	0.261	0.765	**0.343**	**0.794**
**Network_4**	0.163	0.745	0.180	0.699	0.204	0.735	**0.302**	**0.788**
**Network_5**	0.148	0.796	0.174	0.735	0.214	0.769	**0.337**	**0.829**

The highest averaged AUPR and AUROC values are marked in bold for each network. TS, time-series expression data

We also compared PFBNet with iRafNet, HiDi and the winner [34] of the DREAM challenge on the datasets, where all these algorithms adopted different strategies to utilize the prior information from other types of data (e.g., the knockout data). The results are shown in Table 3. As it is shown that, the performance of PFBNet is superior to other three algorithms in terms of both AUPR and AUROC for all the five networks. Specifically, PFBNet achieves 1.4%, 8.5%, 13.3%, 6.7% and **50.3%** improvements than the second-best algorithm in terms of AUPR for the five networks respectively. In addition, the averaged AUROC of PFBNet is 2.1%, 2.5%, 6.1%, 3.5% and 7.8% higher than the second-best algorithm for the five networks respectively. Similar with the results in Table 2, PFBNet achieves better performance in terms of AUPR than that in terms of AUROC. Moreover, the results in Table 3 are much better than that in Table 2, which indicates the importance of fusing the prior information for the GRN inference to some extent.

**Table 3:** Comparison of different methods on the DREAM4 *in*-*silico* size 100 challenge networks

**Method**	**iRafNet**		**HiDi**		**Winner**		**PFBNet**	
**Data used**	TS, KO		TS, KO		KO		TS, KO	
**Metrics**	AUPR	AUROC	AUPR	AUROC	AUPR	AUROC	AUPR	AUROC
**Network_1**	0.552	0.901	0.63	0.916	0.536	0.914	**0.639**	**0.935**
**Network_2**	0.337	0.799	0.448	0.868	0.377	0.801	**0.486**	**0.89**
**Network_3**	0.414	0.835	0.413	0.797	0.39	0.833	**0.469**	**0.886**
**Network_4**	0.421	0.847	0.491	0.852	0.349	0.842	**0.524**	**0.881**
**Network_5**	0.298	0.792	0.251	0.803	0.213	0.759	**0.448**	**0.866**

The highest averaged AUPR and AUROC values are marked in bold for each network. TS, time-series expression data; KO, knockout data

### Performance evaluation on the *E*.*coli* datasets

To further evaluate the performance of our PFBNet algorithm, we also implemented PFBNet and other three algorithms, i.e., GENIE-lag, Jump3 and BiXGBoost on the *E*.*coli* datasets (see the “Datasets” section). It should be noted that, iRafNet and HiDi are ignored as the E.coli datasets only provide the time-series data. In addition, our PFBNet were implemented with the parameters that determined on the DREAM4 challenge datasets. Similarly, averaged AUPR and AUROC were chosen as the criteria in the experiments, and the parameters were set to default values for all algorithms. The comparison results of these algorithms are shown in Table 4, in which we find that the performance of our PFBNet algorithm is also superior to GENIE-lag, Jump3 and BiXGBoost. Especially for the performance in terms of AUPR, PFBNet achieves 33.3%, **120%** and 52.4% improvements than the second-best algorithm for Cold, Heat and Oxidative-stress environments respectively. Meanwhile, PFBNet also achieves the best performance in terms of AUROC, where the average AUROC of PFBNet is 1.2%, 4.5% and 7.5% higher than the second-best algorithm for the three environments respectively. These results suggest that our PFBNet algorithm is also suitable to reconstruct large-scale GRNs from real time-series data.

**Table 4:** Comparison of different methods on the *E*.*coli* datasets (without utilizing the information from prior data)

**Method**	**GEINE-lag**		**Jump3**		**BiXGBoost**		**PFBNet**	
**Data used**	TS		TS		TS		TS	
**Metrics**	AUPR	AUROC	AUPR	AUROC	AUPR	AUROC	AUPR	AUROC
**Cold**	0.011	0.465	0.014	0.535	0.021	0.665	**0.028**	**0.673**
**Heat**	0.012	0.48	0.013	0.513	0.02	0.651	**0.044**	**0.68**
**Oxidative-stress**	0.011	0.458	0.021	0.558	0.018	0.624	**0.032**	**0.671**

The highest averaged AUPR and AUROC values are marked in bold for each network

### Analysis of PFBNet computational complexity

The computational complexity of PFBNet algorithm mainly contains two parts. In the phase of recurrently solving the feature selection subproblems, the GRN inference problem is firstly decomposed into *p* subproblems, where *p* is the number of genes. For each subproblem, the non-linear model of boosting is applied, and the computational complexity for it is *O*(*KDnp*^′^+*np*^′^ log*p*^′^+*knp*^′^), where *n* is the number of samples, *p*^′^≤*p* is the number of the candidate regulators, *K* is the total number of trees, and *D* is the max depth of the tree. The last term of the computational complexity is that of calculating the expression values of the candidate regulators from *k* previous time points. In the phase of fusing the information from prior data, the complexity of the algorithm is *O*(*np*^′^).

In addition, the CPU runtime is another important index for evaluating the GRN inference methods. As PFBNet is an ensemble method based on the boosting, here, we focus on comparing the results with that of BiXGBoost, which also adopts the boosting strategy. The comparison results of BiXGBoost and PFBNet on the three different datasets (DREAM4 InSilico_Size10, DREAM4 InSilico_Size100, and E.coli) are shown in Table 5. These measurements were obtained by using python, an 1.4 GHz Quad-Core Intel Core i5, 8.0GB of RAM memory and a 64-bit Mac operating system. PFBNet takes 2 min 34 s and 51 min to infer DREAM4 InSilico_Size100 and E.coli respectively, which is faster than BiXGBoost’s 6 min 16 s and 3 h 20 min.

**Table 5:** Comparisons of the runtime on different datasets

Method	DREAM4 InSilico_Size100	*E*.*coli*
BiXGBoost	6min 16s	3h 20min
PFBNet	2min34s	51min

## Conclusions

In this study, we develop a novel method, namely PFBNet, to improve the accuracy of GRN inference from time-series expression data by fusing the information of candidate regulators at previous time points as well as the prior information. Specifically, the candidate regulators of *k* time points are taken to construct the regression model, while the decay factor is introduced to calculate the accumulation impact of the candidate regulators of *k* time points. Then, the non-linear model of boosting is applied to solve the feature selection subproblem. In this way, the information of candidate regulators at previous time points is fused in our model, where it is typically ignored by the existing algorithms. Thus, PFBNet would improve the accuracy of the inferred GRN to some extent. Moreover, different with other algorithms (e.g., iRafNet and HiDi) that integrate the prior information in the data preprocessing stage, the prior information is allow to fused into our model by using the statistic technology. The results on the benchmark datasets from DREAM4 challenge and the real *E*.*coli* datasets show that our PFBNet algorithm outperforms significantly other state-of-the-art algorithms, i.e., GENIE-lag, Jump3, BiXGBoost, iRafNet and HiDi.

In the current implementation of our method, for each candidate regulator, the expression data with the gene knocked out should be provided. However, in real application, only a small part of genes is generally knocked-out due to the high cost [23]. Thus, extend our method to fuse the prior information from the expression data with the limited number of genes knocked out would be another interesting way to explore.

## Methods

### The ensemble framework

A GRN can be represented as a directed graph *G*=(*V*,*E*), with a set of directed edges *E* corresponding to regulation relationships and a set of nodes *V* corresponding to genes. Each directed edge *e*_*ij*_∈*E* represents the regulation from gene *i* (i.e., the regulator) to gene *j* (i.e., the target gene). Inferring GRN is to build the *G* from a gene expression matrix *M*=[*X*_1_,⋯,*X*_*p*_] with *p* genes and *N* samples, where the vector *X*_*i*_ denotes the expression values of gene *i* at different samples. As mentioned above, one common practice is to solve the problem under the ensemble framework, where *p* subproblems can be formulated as:

1 $$ X_{i}=f\left(X_{i}^{-}\right)+\epsilon_{i}, i \in(1,2, \cdots, p)  $$

where $X_{i}^{-}$ represents the expression values of candidate regulators (e.g., all genes without gene *i*) of target gene *i*, *f* denotes the selected function (e.g., least angle regression and random forest) that modeling the impact of candidate regulators on the target gene, and *ε*_*i*_ is the random noise. Based on *f*, the confidences of the regulation relationships from each candidate regulator to the target gene could be calculated as the importance of the feature variable. Finally, all regulation relationships from *p* subproblems are ranked according to their confidences, and the top ones are used for constructing the GRN. For time-series data, the subproblem can be formulated as:

2 $$ X_{t}^{i}=f_{t}\left(X_{t^{\prime}}^{-i}\right)+\epsilon_{t^{\prime}}^{i}, i \in(1,2, \cdots, p)  $$

where $X_{t}^{i}$ is the expression value of gene *i* at *t* time point, $X_{t \prime }^{-i}$ is the expression values of candidate regulators of gene *i* at *t*^′^ time point, $\epsilon _{t^{\prime }}^{i}$ indicates the random noise at time *t*^′^.

### Fuse the information from previous time points

In this study, the accumulated impact of the candidate regulators from *k*(*k*=2) previous time points on the target gene at *t* time point is considered; thus, we formulate the subproblem as:

3 $$ X_{t}^{i}=f_{t}\left(U_{k}^{-i}\right)+\epsilon_{t-1}^{i}, i \in(1,2, \cdots, p)  $$

where $U_{k}^{-i}$ denotes a vector collecting the accumulative expression values of the candidate regulators at previous *k* time points, and is defined as:

4 $$ U_{k}^{-i}=f_{a}\left(X_{t-1}^{-i}, \cdots, X_{t-k}^{-i}, \delta\right)  $$

where *f*_*a*_ denotes the accumulation function, 0≤*δ*≤1 is the decay factor as we assume that the impact would be larger if the time point is closer to *t*. In this study, *f*_*a*_ is simply defined as:

5 $$ f_{a}\left(Z_{1}, \cdots, Z_{k}, \delta\right)=\sum_{j=1}^{k} Z_{j} * \delta^{j-1}  $$

It should be noted that, for most of the available algorithms (e.g., GENIE-lag [27]), the *t*^′^ of Eq. (2) is defined to be *t*−1, where it holds the assumption that the expression value of the target gene at *t* time point is only affected by the expression values of the candidate regulators at the previous time point. And the subproblem is defined as:

6 $$ X_{t}^{i}=f_{t}\left(X_{t-1}^{-i}\right)+\epsilon_{t-1}^{i}, i \in(1,2, \cdots, p)  $$

Note that if *k*=1 or *δ*=0, Eq. (3) would be the same as Eq. (6). Therefore, Eq. (6) can be viewed as a special case of our method. The recent method BiXGBoost [25] also considers the impact of candidate regulators from *k* time points, however, it needs to calculate the subproblem for *k* times to select the time point with most impact. Moreover, the final impact of the candidate regulation is more likely to be the accumulation of previous time points rather than the maximal one.

### Non-linear model of boosting

To solve subproblem defined in Eq. (3), a common method for ensemble learning, i.e., boosting is applied here, where it solves the problem via integrating a set of weak learners, and the sum of the all weak learners is viewed as the final prediction. There are various boosting methods, including Gradient Boosting [3], AdaBoost [35], etc. In this study, we introduce a recently developed method, i.e., XGBoost [36] to solve the subproblem and evaluate the importance of the variables. The objective function of XGBoost can be formulated as:

7 $$ \min_{\theta} L^{(t)}(\theta)=\sum_{i=1}^{n}\left[l\left(y_{i}, \widehat{y}_{i}^{(t-1)}\right)+g_{i} f_{t}\left(X_{i} ; \theta\right)+\frac{1}{2} h_{i} f_{t}^{2}\left(X_{i} ; \theta\right)\right]+\Omega\left(f_{t}\left(X_{i} ; \theta\right)\right)  $$

where $\widehat {y_{l}}^{(t)}$ is the prediction value of the target variable of sample *i* at the t-th iteration, *y*_*i*_ is the value of the target variable of sample *i*, *X*_*i*_ denotes a vector collecting all values of the feature variables of sample *i*, *f*_*t*_ is the weak learner integrated at the t-th iteration, *l* is the loss function, *θ* denotes the parameters, $g_{i}=\partial _{\widehat {y}_{l}^{(t-1)}}l\left (y_{i}, \widehat {y}_{i}^{(t-1)}\right)$ and $h_{i}=\partial _{\widehat {y}_{l}^{(t-1)}}^{2}l\left (y_{i}, \widehat {y}_{i}^{(t-1)}\right)$ are the first and second order gradient, and *Ω* is the regularized term as following:

8 $$ \Omega\left(f_{t}\left(X_{i} ; \theta\right)\right)=\gamma T+\frac{1}{2} \lambda\|w\|^{2}  $$

where *T* is the number of leaves in the tree, *γ* and *λ* are the parameters that control the shrinkage, *w* is the leaf weights. The regularization term can smooth the final learnt weights and such that the over-fitting problem would be avoided. The non-linear decision tree is chosen as the base learner, and we apply the number *N*_*i*_ of a feature variable *G*_*i*_ (i.e., candidate regulator) selected to split the target variables among all trees as the importance of *G*_*i*_ on the target gene *j*. That is to say, the confidence of the regulation relationship from gene *i* to gene *j* (i.e., the weight *w*_*ij*_) is evaluated to be *N*_*i*_. The splitting criterion and other details can be referred to [36]. In addition, since each subproblem is solved via the boosting method independently, thus it cannot simply use the confidences of the regulation relationships evaluated from each subproblem for globally ranking. To this end, we employ a L2-norm based normalization to solve this problem. And the weights *w*_*ij*_ for each subproblem are normalized as:

9 $$ \widetilde{w_{i j}}=\frac{w_{i j}}{\sqrt{\sum_{i=1}^{p} w_{i j}^{2}}}, i \neq j  $$

where *p* is the number of candidate regulators for gene *j* in each subproblem.

### Fuse the information from prior data

Since other types of data (e.g., the gene expressions from knockout experiments) often provide more information about the directionality of regulatory relationships, it is important to integrate these data for inferring the more reliable and accurate GRN. Some representative algorithms, (e.g., HiDi and iRafNet) typically integrate the prior information supported by other types of data in process of data preprocessing. Here, we present a way that fuse the prior information into our model (Other types of data could be defined in the similar way).

Without loss of generality, we denote $x_{ij}^{KO}$ the expression of gene *j* after knockout the gene *i*, and the prior information $I_{i j}^{K O}$ is defined as:

10 $$ I_{i j}^{K O}= \left\{ \begin{aligned} \frac{\left|x_{i j}^{K O}-\overline{x_{i j}^{K O}}\right|}{\sigma_{i j}^{K O}}, & \quad i \neq j, p_{r}=1 \\ 1 \quad\quad\quad, &\quad p_{r}=0\\ \end{aligned} \right.  $$

where *p*_*r*_ is the control parameter that determines whether or not to fuse the prior data information (i.e., 1: fuse; 0: not fuse) and $\overline {x_{i j}^{K O}}$ is the averaged expression of gene *j* for all knockout experiments, and $\sigma _{ij}^{KO}$ is the standard deviation of the expression values of gene *j* for all knockout experiments.The value of $I_{ij}^{KO}$ reflects the significant levels of changes of $x_{ij}^{KO}$ among all knockout experiments(see Table S2). The larger the value, the more significant it is. Meanwhile, we hold the assumption that if the $I_{ij}^{KO}$ is large, the confidence of the regulation relationship that from gene *i* to gene *j* would be high. To this end, we apply $I_{ij}^{KO}$ to update the global confidences of all regulation relationships *w*_*ij*_ as:

11 $$ \widehat{w_{i j}}=w_{i j} * I_{i j}^{K O}, i \neq j  $$

where *w*_*ij*_ denotes the confidence of the regulation relationship from gene *i* to gene *j*, and it is calculated through the feature importance evaluation of the boosting method. It should be noted that other types of data (e.g., the knock down data) may also be integrated in the similar way. Additionally, we also use a statistical technique to further update the weights *w*_*ij*_, where it is based on the hypothesis that if a candidate regulator *i* regulates multiple target genes, it would be an important regulator and the confidences of all regulation relationships about this gene should be elevated. In the light of this, we formulate the update of the weights *w*_*ij*_ for each candidate regulator *i* as:

12 $$ \overline{w_{ij}}=\sigma_{i}^{2} * w_{i j}, j=1,2,...,p, j \neq i  $$

where $\sigma _{i}^{2}$ denotes the variance of all *w*_*ij*_ for candidate regulator *i*. Note that the GRN is often sparse, thus the values of most *w*_*ij*_ for candidate regulator *i* would be small. Therefore, if the value of $\sigma _{i}^{2}$ is relatively large, it would mean that the confidences of several regulation relationships about candidate regulator *i* are large, such that candidate regulator *i* is likely to regulate those corresponding target genes.

## Supplementary information

**Additional file 1** Additional Fig S1. The AUPR and AUROC of PFBNet with different *subsample* value on DREAM4 inSilico_Size100. The averaged AUPR and AUROC were chosen as the criteria in the experiments, and the *subsample* is set from 0.1 to 1 with step 0.1.

**Additional file 2** Additional Fig S2. The AUPR and AUROC of PFBNet with different *colsample*_*bytree* value on dREAM4 inSilico_Size100. The averaged AUPR and AUROC were chosen as the criteria in the experiments, and the *colsample*_*bytree* is set from 0.1 to 1 with step 0.1.

**Additional file 3** Additional Fig S3. The AUPR and AUROC of PFBNet with different *learning*_*rate* on dREAM4 inSilico_Size100. The averaged AUPR and AUROC were chosen as the criteria in the experiments, and the *learning*_*rate* is set from 0.0001 to 0.001 with step 0.0001.

**Additional file 4** Additional Fig S4. The AUPR and AUROC of PFBNet with different *max*_*depth* on dREAM4 inSilico_Size100. The averaged AUPR and AUROC were chosen as the criteria in the experiments, and the *max*_*depth* is set from 1 to 9 with step 1.

**Additional file 5** Additional Fig S5. The AUPR and AUROC of PFBNet with different *min*_*child*_*weight* on dREAM4 inSilico_Size100. The averaged AUPR and AUROC were chosen as the criteria in the experiments, and the *min*_*child*_*weight* is set from 1 to 9 with step 1.

**Additional file 6** Additional Fig S6. The AUPR and AUROC of PFBNet with different *gamma* values on dREAM4 inSilico_Size100. The averaged AUPR and AUROC were chosen as the criteria in the experiments, and the *gamma* value is set from 0.1 to 0.9 with step 0.1.

**Additional file 7** Additional Table S1. An example of fusing the prior information from previous time points.

**Additional file 8** Additional Table S2. An example of fusing the information from Knock out data.

## Data Availability

All relevant data in this study as well as our Python implementation of PFBNet are available at https://github.com/ddche/PFBNet.

## Abbreviations

ARACNE: algorithm for the reconstruction of accurate cellular NEtworks
AUPR: an area under the precision-recall curve
AUROC: an area under the receiver operating characteristic curve
CLR: context likelihood of relatedness
DREAM: Dialogue for reverse engineering assessments and methods
GENIE3: gene network inference with ensemble of trees
GRN: Gene regulatory network
BiXGBoost: Bidi-rectional XGBoost
Jump3: gene network inference with jump trees
HiDi: an efficient reverse engineering schema for large-scale dynamic regulatory network reconstruction
iRafNet: integrative random forest for gene regulatory network PFBNet: Priori-fused boosting network inference method
