# Supplementary figures and images for: PFBNet: a priori-fused boosting method for gene regulatory network inference
Source: BMC Bioinformatics. 2020 Jul 14;21:308. doi: 10.1186/s12859-020-03639-7 (PMC7362553; doi:10.1186/s12859-020-03639-7)

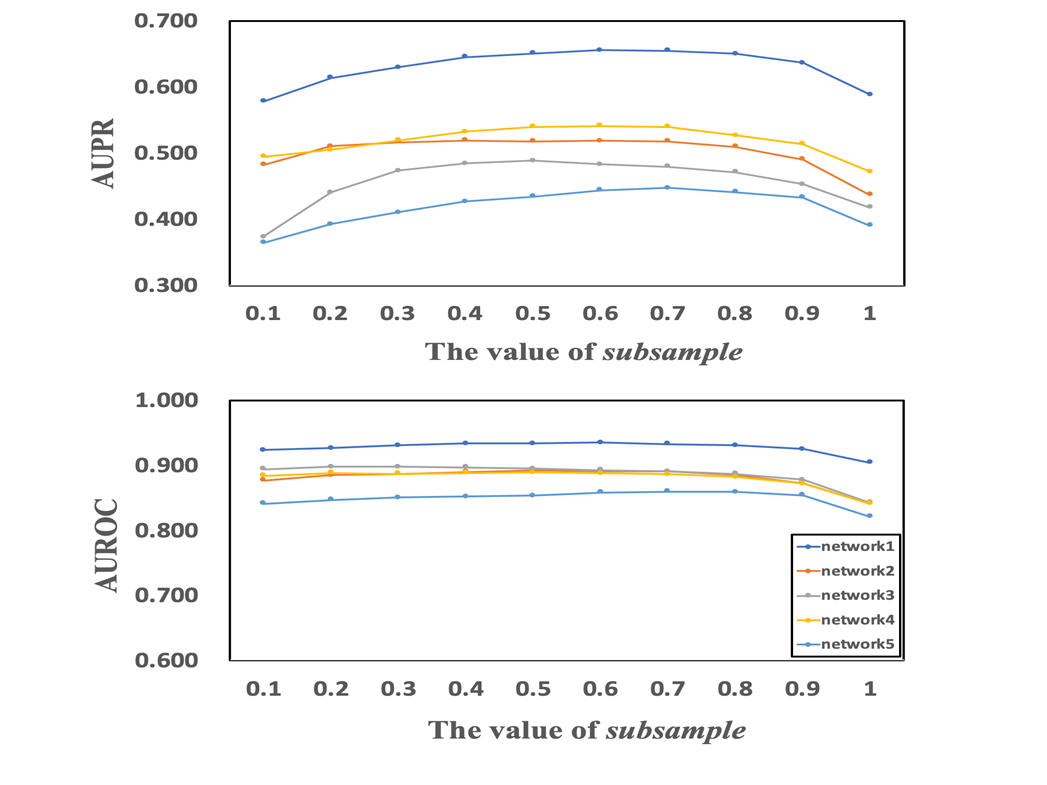

Supplement: Supplementary file 1 — Additional file 1 Additional Fig S1. The AUPR and AUROC of PFBNet with different subsample value on DREAM4 inSilico_Size100. The averaged AUPR and AUROC were chosen as the criteria in the experiments, and the subsample is set from 0.1 to 1 with step 0.1. [file 12859_2020_3639_MOESM1_ESM.tif]

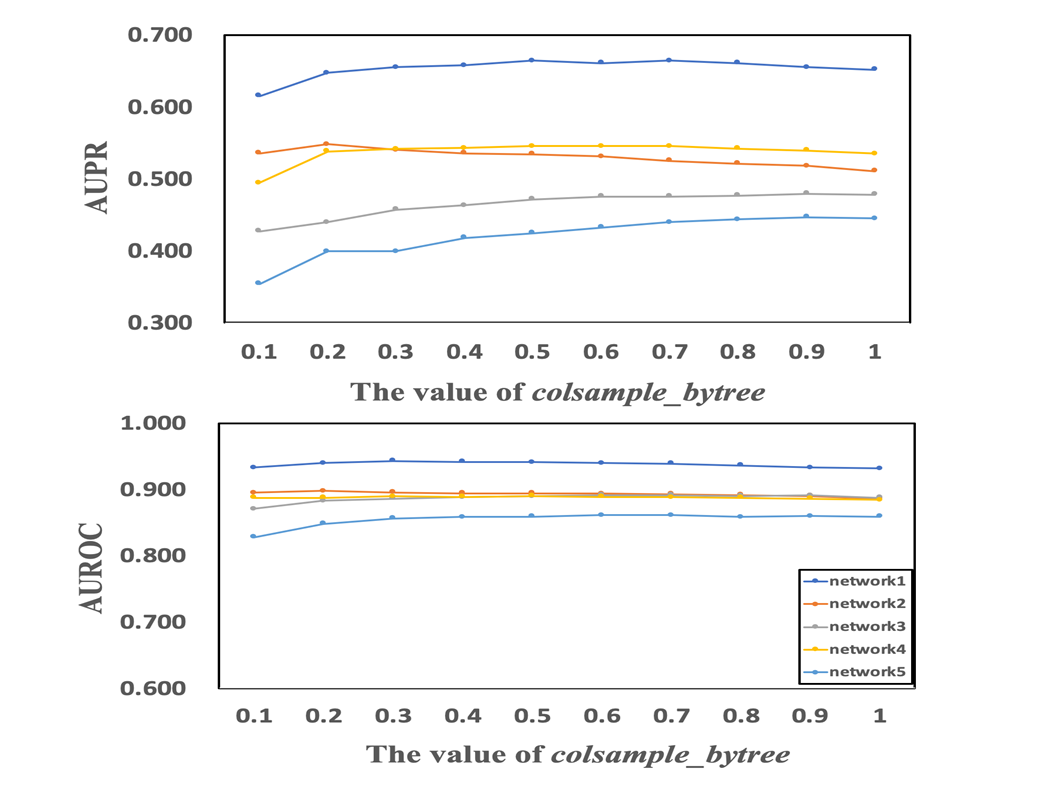

Supplement: Supplementary file 2 — Additional file 2 Additional Fig S2. The AUPR and AUROC of PFBNet with different colsample_bytree value on dREAM4 inSilico_Size100. The averaged AUPR and AUROC were chosen as the criteria in the experiments, and the colsample_bytree is set from 0.1 to 1 with step 0.1. [file 12859_2020_3639_MOESM2_ESM.tif]

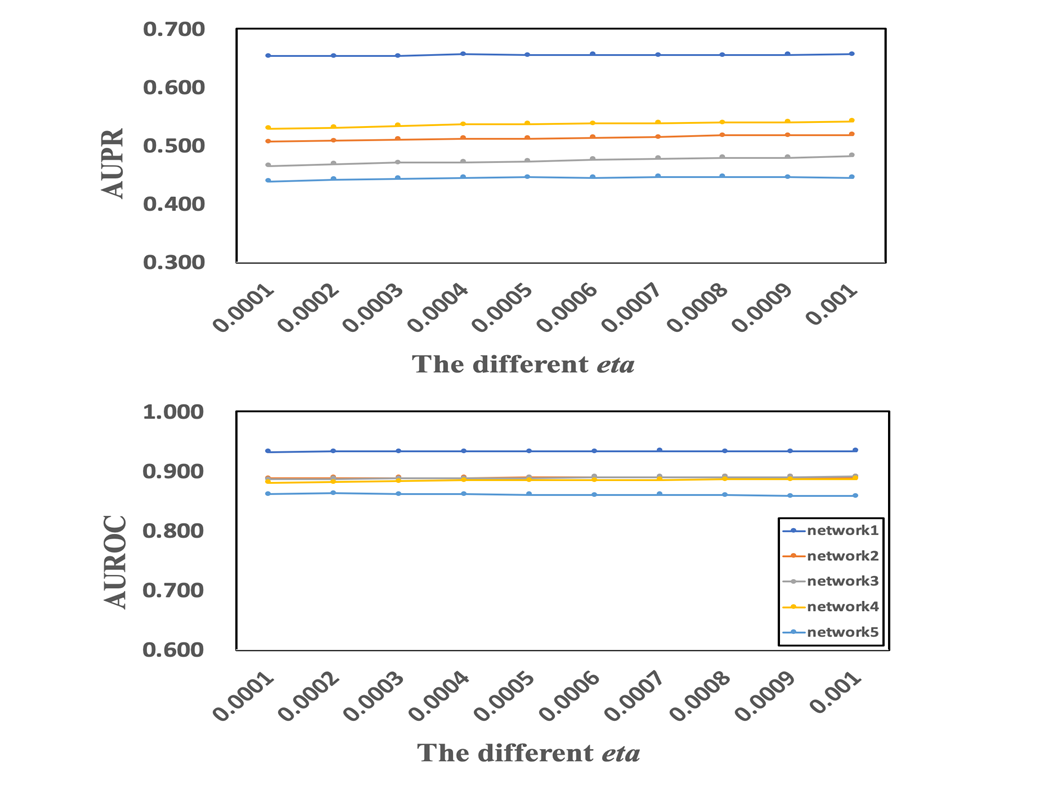

Supplement: Supplementary file 3 — Additional file 3 Additional Fig S3. The AUPR and AUROC of PFBNet with different learning_rate on dREAM4 inSilico_Size100. The averaged AUPR and AUROC were chosen as the criteria in the experiments, and the learning_rate is set from 0.0001 to 0.001 with step 0.0001. [file 12859_2020_3639_MOESM3_ESM.tif]

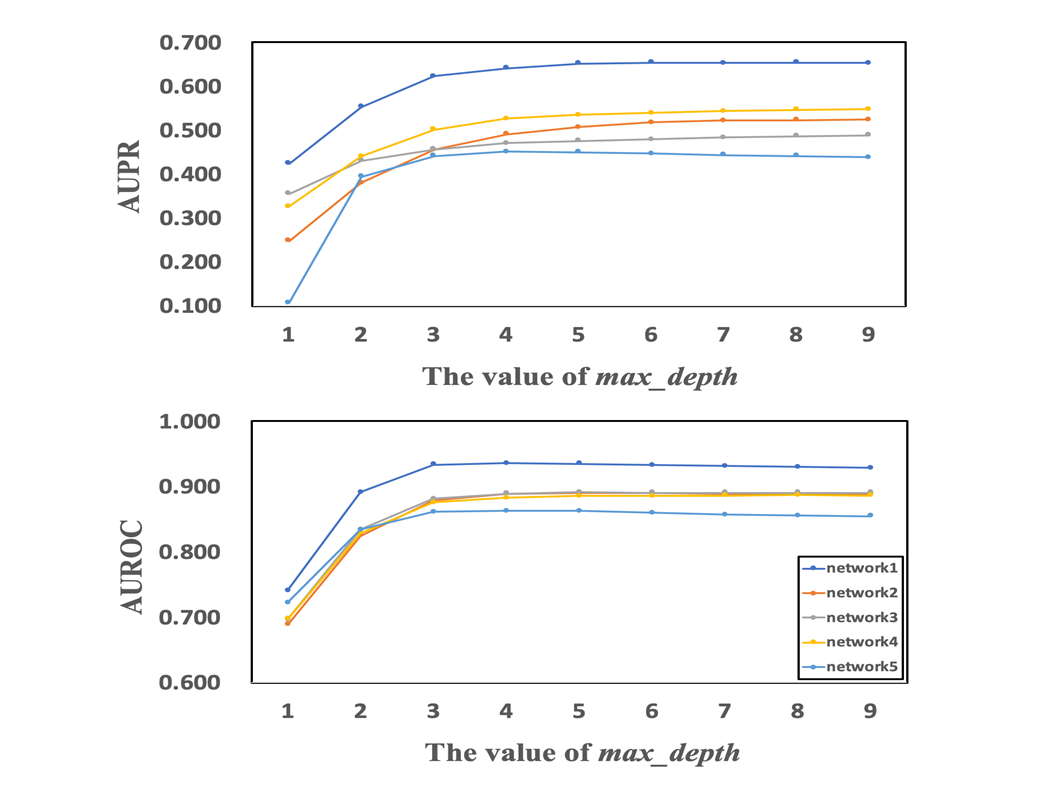

Supplement: Supplementary file 4 — Additional file 4 Additional Fig S4. The AUPR and AUROC of PFBNet with different max_depth on dREAM4 inSilico_Size100. The averaged AUPR and AUROC were chosen as the criteria in the experiments, and the max_depth is set from 1 to 9 with step 1. [file 12859_2020_3639_MOESM4_ESM.tif]

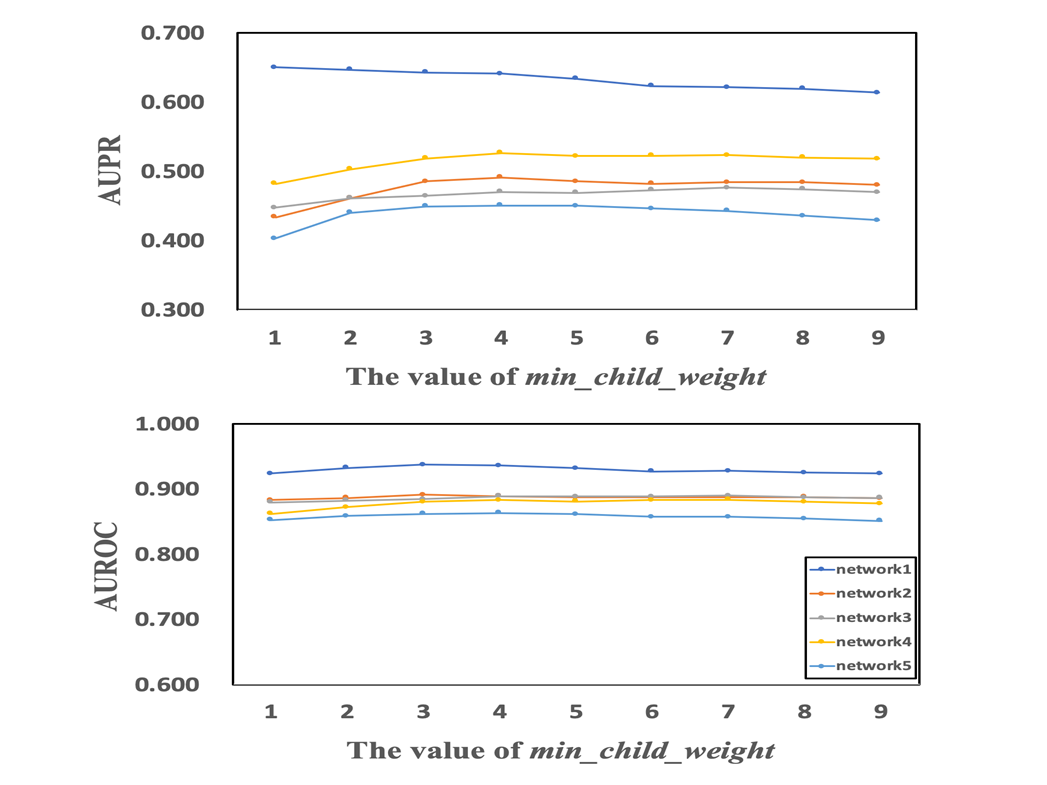

Supplement: Supplementary file 5 — Additional file 5 Additional Fig S5. The AUPR and AUROC of PFBNet with different min_child_weight on dREAM4 inSilico_Size100. The averaged AUPR and AUROC were chosen as the criteria in the experiments, and the min_child_weight is set from 1 to 9 with step 1. [file 12859_2020_3639_MOESM5_ESM.tif]

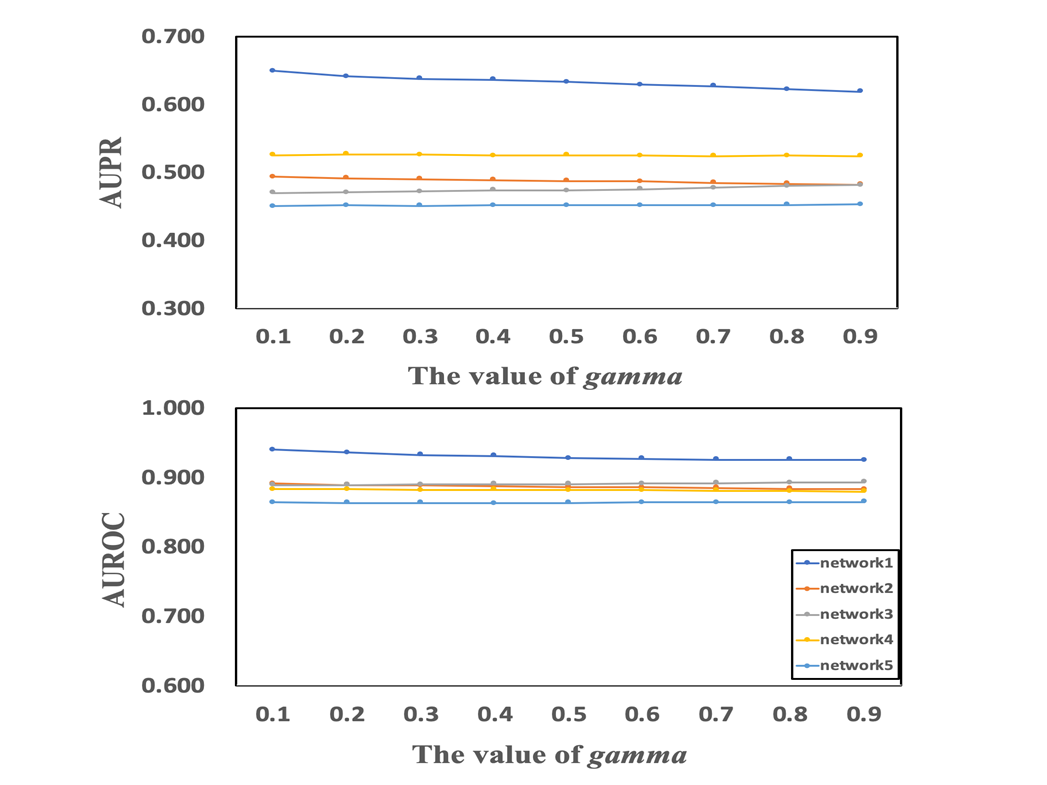

Supplement: Supplementary file 6 — Additional file 6 Additional Fig S6. The AUPR and AUROC of PFBNet with different gamma values on dREAM4 inSilico_Size100. The averaged AUPR and AUROC were chosen as the criteria in the experiments, and the gamma value is set from 0.1 to 0.9 with step 0.1. [file 12859_2020_3639_MOESM6_ESM.tif]
