# Supplementary material for: PFBNet: a priori-fused boosting method for gene regulatory network inference
Source: BMC Bioinformatics. 2020 Jul 14;21:308. doi: 10.1186/s12859-020-03639-7 (PMC7362553; doi:10.1186/s12859-020-03639-7)
Supplement: Supplementary file 7 — Additional file 7 Additional Table S1. An example of fusing the prior information from previous time points. [file 12859_2020_3639_MOESM7_ESM.docx]

Table S1. An example of fusing the prior information from previous time points

| Sample  ID | Target gene expression  (t time point) | Candidate regulator expression  (t-1 time point) | Candidate regulator expression  (t-2time point) |
| --- | --- | --- | --- |
| 1 | $x_{t}^{11}$ | $x_{t-1}^{21}, x_{t-1}^{31},\cdots, x_{t-1}^{p1}$ | $x_{t-2}^{21}, x_{t-2}^{31},\cdots, x_{t-2}^{p1}$ |

In this study, the number of the prior time points that taken in consideration is one main parameter of our PFBNet (i.e., parameter *k*), where it was tuned on the DREAM4 challenge datasets and set to **2** (see Section Parameters setting of PFBNet). $x_{t}^{ij}$ denotes the expression of $i^{th}$ gene at t time point on sample j. Thus, the subproblem(related with gene 1) for sample 1 is formulated as $x_{t}^{i1}=f\left( x_{t-1}^{21}+x_{t-2}^{21}*\delta; x_{t-1}^{31}+x_{t-2}^{31}*\delta; \cdots;x_{t-1}^{p1}+x_{t-2}^{p1}*\delta\right)$.
