# Supplementary material for: PFBNet: a priori-fused boosting method for gene regulatory network inference
Source: BMC Bioinformatics. 2020 Jul 14;21:308. doi: 10.1186/s12859-020-03639-7 (PMC7362553; doi:10.1186/s12859-020-03639-7)
Supplement: Supplementary file 8 — Additional file 8 Additional Table S2. An example of fusing the information from Knock out data. [file 12859_2020_3639_MOESM8_ESM.docx]

Table S2. An example of fusing the information from knock out data

| Expression of gene 1 after knockout gene 2 | Expression of gene 1  after knockout gene 3 | … | Expression of gene 1  after knockout gene p |
| --- | --- | --- | --- |
| $x_{21}^{KO}$ | $x_{31}^{KO}$ | … | $x_{p1}^{KO}$ |

For gene 1, we can obtained the related information (i.e., the expressions of gene 1 after knockout the respective genes) from the knockout data. Then, $I_{i1}^{KO}, i=2,3,\ldots, p$ can be calculated according to Eq. (10), where $\overline{x_{i1}^{KO}}$ and $\overline{\sigma_{i1}^{KO}}$ are the mean value and the standard deviation of these expression values. If the value of $I_{i1}^{KO}$ is relatively large (e.g., >1), it means that the expression of gene1 varied relatively significantly after knockout gene$1$ as compared with that of knockout other genes. In other words, gene$1$ is most likely regulated by gene $i$. Ideally, the more likely it is, the larger value of $I_{i1}^{KO}$ is. In the light of this, we elevate the value of $w_{i1}$ by multiplying $I_{i1}^{KO}$ (large value). On the other hands, the value of $w_{i1}$ would be decrease if $I_{i1}^{KO}$ is relatively small (e.g., < 1). In this way, all $w_{ij}$ would be update.
